# CAR T cell therapy in solid tumors: A review of current clinical trials

**DOI:** 10.1002/jha2.356

**Published:** 2021-12-07

**Authors:** Urvi Patel, John Abernathy, Bipin N Savani, Olalekan Oluwole, Salyka Sengsayadeth, Bhagirathbhai Dholaria

**Affiliations:** ^1^ College of Pharmacy The University of Tennessee Health Science Center Nashville Tennessee USA; ^2^ College of Medicine The University of Tennessee Health Science Center Memphis Tennessee USA; ^3^ Department of Hematology‐ Oncology Vanderbilt‐Ingram Cancer Center Vanderbilt University Medical Center Nashville Tennessee USA

**Keywords:** CAR T cell therapy, clinical trials, solid tumors

## Abstract

Chimeric antigen receptor (CAR) T cell therapy has made tremendous strides in the arena of hematological malignancies with approved therapies in certain leukemias, lymphomas, and recently myeloma with overall highly favorable response rates. While numerous clinical studies are still ongoing for hematological malignancies, research is developing to translate the feasibility of CAR T therapy in solid organ malignancies. Unfortunately, the majority of diagnosed cancers are primarily solid tumors. Thus, a highly unmet clinical need for further research and development exists in this field. This review article highlights currently active clinical trials and a few pertinent preclinical studies involving CAR T cell therapy in solid tumors while briefly discussing study outcomes and potential key targets that may allow for the feasibility of this therapy option. Finally, we mention critical challenges existing in the solid tumor environment and discuss developing strategies that may potentially overcome the existing barriers to CAR T cell progress in solid tumors.

## 1 BACKGROUND

CAR T cells are a form of genetically engineered, patient‐ or donor‐derived immune cells that are designed to express recombinant or chimeric antigen receptors on their surface to recognize and target specific tumor‐associated antigens and induce cell‐mediated attack that leads to tumor cell death. This ability to reprogram T cells has enabled novel opportunities to personalize cancer therapy, particularly for hematologic malignancies, leading to five approved CAR T cell therapies and more in the pipeline. In addition, with the record success of CAR T cells in cancers such as leukemia and lymphoma, a growing number of clinical trials are underway focusing on translating this type of therapy option to solid tumors.

## 2 INTRODUCTION

Current United States Food and Drug Administration (FDA)‐ approved CAR T therapies primarily target the B cell lineage antigen CD19 except the latest approved this year, idecabtagene vicleucel (Abecma^®^), which targets the B‐cell maturation antigen (BCMA) and is the first novel immunotherapy indicated for relapsed or refractory multiple myeloma. In addition, lisocabtagene maraleucel (Breyanzi^®^) also received FDA approval as a CD19 targeted gene therapy for refractory or relapsed diffuse large B cell Lymphoma (DLBCL) in February 2021. Other approved CD19‐targeted CAR T therapies include tisagenlecleucel (Kymriah^®^), axicabtagene ciloleucel (Yescarta^®^), and brexucabtagene autoleucel (Tecartus^®^). These CD19‐targeted therapies have become important treatment options for patients with acute B cell lymphoblastic leukemia (B‐ALL) or certain aggressive B cell non‐Hodgkin lymphomas (NHLs) and have been able to induce complete remissions in heavily pretreated patients with the extensive disease [1, 2, 3, 4].

While these few trials have rendered remarkable success in bringing these novel therapies to market, numerous other trials are continuing in the arena of hematologic malignancies. This potentiates the opportunity to explore the novelty of CAR T cell therapy in solid tumors. Current studies of CAR T cells in solid tumors primarily evaluate safety outcomes and report preliminary research findings thus far. Because the primary and secondary outcomes data continue to evolve for these trials, this article focuses on delivering a brief overview, reviewing essential targets, and discussing the current clinical trials of CAR T cell therapy in solid tumors.

## 3 BRAIN CANCER

Glioblastoma (GBM) is among the most common forms of malignant primary brain tumors. Current treatment options typically consist of surgery followed by chemotherapy or radiotherapy with a median 2‐year patient survival rate of less than 30% [5]. In addition, because of its complexity, current treatments do not provide adequate disease control for patients [5]. Immunotherapy with CAR T cells is being studied as a novel option for this disease [6]. In part to investigate potential targets for CAR T cell therapy in GBM, clinical trials are exploring a variety of immunotherapeutic strategies, one of which is the target IL13Ra2, a commonly expressed membrane‐bound protein in over 75% of GBMs that is associated with activating the mammalian target of rapamycin (mTOR) pathway favoring tumor growth [7].

One ongoing phase 1 clinical trial investigates the safety, efficacy, and feasibility of IL13Ra2 as a potential CAR T‐cell target [8] in patients with recurrent or refractory malignant GBM due to its specificity for GBM tumor cells and limited expression on normal brain cells (NCT02208362). The findings from a patient case report related to this trial demonstrated a transient complete response when given intraventricular CAR T cells targeting IL13Rα2, with a clinical response sustained for 7.5 months after therapy initiation and improvements in quality of life [9].

Another considerable CAR T cell target is the human epidermal growth factor receptor 2 (HER2), a tyrosine kinase receptor that is overexpressed in GBM and many other human cancers [10]. One study evaluates the safety of autologous CAR T cells targeting HER2 in subjects with progressive recurrent or refractory HER2‐positive primary central nervous system (CNS) tumor or HER2 positive tumor metastatic to the CNS after standard care interventions (NCT02442297). So far, preliminary findings are not conclusive, but this trial is ongoing. Epidermal growth factor receptor variant III (EGFRvIII) is another target generated via molecular alterations and is a tumor‐specific protein present in 25‐30% of newly diagnosed GBMs, making it another potential option for CAR T cells [11]. A recently completed phase 1 study combined EGFRvIII‐directed CAR T cells with pembrolizumab, a programmed cell death protein 1 (PD‐1) inhibitor. This open‐label study assessed the safety and tolerability of EGFRvIII specific CAR T cells in combination with pembrolizumab in newly diagnosed EGFRvIII+, MGMT (o‐methylguanine‐DNA methyltransferase) ‐unmethylated GBM in seven patients (NCT03726515). Results from this trial are pending. Of note, one additional target was identified that shows preclinical favorability for the tumor differentiation antigen mesothelin (MSLN) for its overexpression in select solid tumors, including brain tumors [12].

## 4 COLORECTAL CANCER

Several early experiences with CAR T cell therapy in colorectal cancer include phase I trials investigating targets such as transmembrane 4 L six family member 1 (TM4SF1) and epithelial cell adhesion molecule (EpCAM), which are both highly expressed in many epithelial‐derived solid tumors.

The carcinoembryonic antigen (CEA) is a classic tumor marker overexpressed in more than 80% of colorectal cancer (CRC) patients. However, CEA is being investigated for other solid tumors as well. An open‐label phase 1 trial is evaluating the efficacy and safety of CEA‐targeted CAR T cell therapy in relapsed or refractory CEA+ cancers (NCT04348643) assessing cancers like CRC. Most of these trials include CRC as one of the cancers of inclusion but eligibility is not limited to CRC alone. However, one early phase 1 study first posted in August 2020 focused on CRC alone. It measures the outcomes such as adverse events related to CEA‐specific CAR T cells, circulating tumor cells after therapy, and maximum tolerated doses (NCT04513431).

An additional trial of CEA‐targeted CAR T cells is ongoing for metastatic CRC in conjunction with other CEA + tumors (NCT03682744). So far in this trial, severe adverse effects of CAR T cell therapy have not been reported yet they persisted in circulation for only a few days to a few weeks, with all patients having undetectable levels via PCR 4‐6 weeks post‐CAR‐T infusion (NCT03682744).

After some observations from these clinical trials, investigators find it essential to mention the potential for on‐target off‐tumor toxicity concerning CEA since it is present on various epithelial cells in multiple organs. However, to mitigate the potential for such toxicity, some studies evaluate the direct administration of CAR T cells via the hepatic artery. Yet, the data recorded from trials investigating this approach is limited [13].

A patient case report observes how anti‐CEA CAR‐T cells were infused via the hepatic artery using pressure‐enabled drug delivery (PEDD) technology and was not associated with any serious or on‐target off‐tumor adverse events. Following the CAR‐T treatment, a complete metabolic response within the liver was sustained for 13 months revealed by positron emission tomography and normalized serum tumor markers with an abundance of CAR+ cells found within post‐treatment tumor specimens [14]. Further studies will investigate this unique delivery method in the treatment for patients with liver metastases.

Another phase 1 trial is assessing the safety and tolerability of CYAD‐101, a CAR‐ T receptor encoding natural killer group 2D (NKG2D) receptor within its intracellular domain. Data from preclinical models determined NKG2D to be a commonly over‐expressed target in CRC. In this trial, among fifteen patients with unresectable metastatic CRC receiving three doses of CYAD 101 cells after standard chemotherapy, two patients had a partial response and nine were of stable disease [15].

## 5 PANCREATIC CANCER

While current immunotherapy with antibodies targeting PD‐1, PD‐L1, and CTLA‐4 are used as treatments in pancreatic cancer, CAR T cell therapy is a particularly appealing and emerging therapy consideration for this disease. Pancreatic ductal adenocarcinoma (PDAC) accounts for more than 90% of pancreatic cancer cases and has a poor prognosis with limited response to current treatments. Because PDAC is a highly aggressive and fatal malignancy, it has become among the more common types of pancreatic cancers studied in clinical trials.

Existing preclinical and clinical studies data show several antigen targets as potential CAR T cell therapy candidates in pancreatic cancer. For instance, preclinical work in pancreatic cancer targeting the overexpressed tumor glycoprotein mesothelin has led to human studies. The most experience with CAR T cells in PDAC patients has been with targeting mesothelin since approximately 80% of pancreatic carcinomas express mesothelin and, as a result, has become a key target for CAR T cell therapy trials [16],[17] (NCT03323944).

In a phase I study of HER2‐directed CAR T cells in advanced pancreatic cancers [18], measured outcomes include off‐tumor toxicities of HER2, partial response rate, achievement of stable disease, and therapeutic levels of HER2 CAR T cells in vivo. Of the eleven patients enrolled, preliminary findings show one case of febrile syndrome and transaminitis during the infusion and a case of upper gastrointestinal hemorrhage post‐infusion. However, for clinical efficacy, one patient received a partial response after 4.5‐months and 5 achieved stable disease [18].

Another ongoing phase 1 trial is studying the prostate stem cell antigen (PSCA) directed CAR T cells in PSCA+ metastatic pancreatic cancer and evaluating feasibility, safety, and clinical activity of PSCA‐specific CAR‐T cells (NCT02744287). Other targets being evaluated in preclinical studies include CEA, Claudin18.2, and MUC1 ([19, 20]).

A more recent study evaluated the potential efficacy of a new target, and identified a novel target, carcinoembryonic antigen‐related cell adhesion molecule (CEACAM7) for PDAC tumors, and demonstrate that CEACAM7‐directed CAR T cells can effectively mediate remission of late‐stage patient‐derived PDAC xenograft tumors based on their research of in vitro and in vivo models [21].

## 6 RENAL AND HEPATIC CANCERS

Several early‐stage trials involving CAR T cells for renal cell carcinoma (RCC) are ongoing, assessing the feasibility of mainly two receptor tyrosine kinase targets: AXL, which plays diverse roles in tumor cell proliferation, migration, and survival (NCT03393936) and ROR2 (NCT03960060). These targets are under investigation for their safety and efficacy in an open‐label, two‐arm phase I/II trial in adult subjects with relapsed and refractory stage IV metastatic RCC (NCT03393936).

Another potential antigen target in hepatocellular carcinoma (HCC) for CAR T is glypican‐3 (GPC3). Two prospective phase I studies in adults with advanced GPC3+ HCC used infusion of GPC3 targeting CAR T cell after cyclophosphamide and fludarabine‐based lymphodepletion [22]. Out of 13 patients, nine experienced CRS and no patients experienced grade 3 or grade 4 neurotoxicity. The overall survival rates at 3 years were 10.5%, 1 year was 42%, and 6 months was 50.3%. One patient from the trial with the sustained stable disease was alive after 44.2 months. In addition, clinical trials are also investigating the use of GPC3 CAR T in combination therapy with checkpoint inhibitors, particularly with PD‐L1‐positive HCC [23].

A different phase I study used CD133 targeting CAR T in advanced metastatic solid tumors [24]. While enrollees that qualified had a variety of cancers, 23 had HCC and made up most recruited patients. Three of the HCC patients achieved partial remission and 14 achieved stable disease. The 3‐month disease control rate was 65.2% and the median progression‐free survival was 5 months. Subsequent CAR T infusions were noted to provide a longer period of disease stability, particularly in patients that experienced tumor reduction following the first infusion.

Liver cancers also express CD147, a type I transmembrane glycoprotein, that has been found in other cancers such as breast cancer, lung cancer, and bladder carcinoma [23]. CD147 has been shown to promote tumor progression, invasion, and metastasis through the stimulation of the secretion of matrix metalloproteinase. NCT03993743 is a phase I study investigating CD147‐targeting CAR T in advanced HCC. The CAR T used included a novel inducible control system called Tet‐On that can reversibly activate or inactive the CAR gene expression when doxycycline (Dox) is present or absent. Xenograft experiments with Dox+ Tet‐CD147 CAR T treatment of HCC showed effective inhibition of growth of cancer cells and demonstrated regulation by Dox administration both in vitro and in vivo.

## 7 PROSTATE CANCER

Like pancreatic cancer, PSCA is a commonly found antigen in prostate cancer, making it a feasible target to consider for CAR T cell therapy. For example, one phase I CAR T cell trial studies PSCA in patients with PSCA+ metastatic castration‐resistant prostate cancer (mCRPC) and measures outcomes such as safety, tolerability, and recommended phase II dose (NCT03873805). In addition, comparable results are being measured in another study evaluating escalation doses of CAR T cells designed to target the prostate‐specific membrane antigen (PSMA), a cell surface antigen found on prostate cells along with other epithelial surfaces and plays a vital role in prostate cancer (NCT04249947).

A phase I trial for anti‐PSMA CAR T indicated that interleukin‐2 (IL‐2) may play a role in the success of CAR T tumor destruction [25]. Enrollees in this clinical trial were treated with lymphodepletive chemotherapy after which they received dose‐escalating CAR T with a continuous infusion of low dose IL2 with the goal of ≥ 20% CAR T engraftment. The goal for engraftment was achieved in three of the five enrollees. Surprisingly, IL2 was depleted in an inverse correlation with the CAR‐T activation. No toxicities or anti‐CAR reactivities were observed. Two of the five patients achieved partial responses and PSA declines of 50% and 70%. A third patient had a minor response to therapy. The responses were assessed as being unrelated to dose size, inversely correlated with engraftment, and directly correlated with plasma IL2.

Consideration has been given to the application of focal CAR T instead of using alternative focal treatments currently available such as high‐intensity focused ultrasound and focal laser ablation [26]. Focal therapy is not recommended with standard treatment protocols due to a lack of long‐term studies. Additionally, prostate tumors can be multifocal in which case focal treatment would not completely eliminate the tumor if a lesion was missed. However, CAR T could be an effective focal treatment alternative if locally administered and would continue to seek out other cancerous lesions to eliminate the lesions. Proposals for how to focally administer CAR T have been made with consideration to guiding injection via reprogrammed MRI‐TRUS fusion‐based robotic biopsy systems to reach cancerous lesions and allow for a biopsy of patients simultaneously.

While other studies are in progress, most of these early‐phase clinical trials primarily evaluate these two antigens in different contexts, such as other subtypes of prostate cancer as well as other solid tumor categories. However, the data are limited to draw accurate summaries of their progress thus far warranting further exploration for future studies.

## 8 OVARIAN CANCER

The research on CAR T cell therapy in ovarian cancer is relatively limited. However, a recent phase 1 clinical trial has been published assessing the safety of CAR T cells specific for alpha‐folate receptor (FR) for the treatment of metastatic ovarian cancer. While only a few potential receptor targets have been researched for ovarian tumors, FR was studied in this trial. FR CAR T cells were peripherally administered to patients with FR + ovarian cancer refractory to platinum/paclitaxel‐based chemotherapy [27].

The trial results showed no reduction in tumor burden and lacked specific localization of CAR T cells to the tumor, followed by toxicities related to IL‐2 activation. In addition, radiographic CAR T labeled imaging and PCR analysis showed that the large volume of CAR T cells began to decline rapidly after 2 days of infusion and further regressed to undetectable levels by 1 month. To improve site localization, another clinical trial is utilizing direct peritoneal administration of FR+ targeted CAR T cells and evaluating its safety and feasibility with or without lymphodepletion therapy (NCT03585764).

Another phase 1 trial has been targeting detectable levels of the mucin 16 (MUC16) antigen. This protein is present in about 70% of ovarian tumors, and the trial evaluates the safety of different CAR T cell concentrations after standard chemotherapy and their effects on cancer [28] (NCT02498912).

## 9 BREAST CANCER

As mentioned earlier, MSLN is a target seen in various solid tumors and serves as a potential therapeutic target in breast cancer. An ongoing phase 1 clinical trial is assessing the safety and tolerability of MSLN‐specific CAR T cells in patients with metastatic mesothelin‐expressing breast cancer (NCT02792114), while another study is investigating HER2‐specific CAR T cells to target the commonly found receptor human epidermal growth factor receptor 2 (HER2) in breast cancer along with other HER2 positive tumors (NCT02442297; NCT03696030).

Finally, it is worth noting one other potential target: the cell‐surface molecule c‐Met expressed in 50% of breast cancers with low levels of expression on healthy tissue. Two studies ([29],[30]) have identified the molecule as a CAR T cell therapy target. Therefore, they may initiate clinical trials to investigate the safety and feasibility of this target while considering the limit of on‐target off‐tumor toxicity that may ensue.

## 10 THORACIC CANCER

Thus far, CAR T cell trials for thoracic cancers have focused primarily on malignant pleural mesothelioma and (MPM) (NCT02414269) and advanced‐stage non‐small cell lung cancer (NSCLC) (NCT02706392). These clinical trials are currently in phase 1 and are investigating the safety of these targets in lung cancer and other solid tumors where applicable.

A wide variety of targets are currently being evaluated for CAR T cell therapy in lung cancer and include EGFR, HER2, MSLN, MUC1, CEA, ROR1, and PD‐L1, several of which were discussed previously with other solid tumors. Among these, EGFR and MSLN specific CAR T cells seem to be more promising compared to others due to the antigen's higher specificity and lower on‐target, off‐tumor toxicity concern.

One open‐label phase I investigated the use of regional delivered autologous mesothelin‐targeted CAR T with pembrolizumab for MPM [31]. In this study, 27 patients with MPM received intrapleural mesothelin targeting CAR T; 18 patients received CAR T and pembrolizumab. Outcome results were based on 23 patients and 18 combination therapy patients. The median overall survival following CAR T treatment was 23.9 months and 1‐year survival was 83%. Radiologic imaging showed that the best overall response was a partial response in two of 16 (12.5%) patients, stable disease in 9 of the 16 patients (56.3%), and progressive disease in 5 0f 16 (31.3%) of enrollees. Another study, NCT02414269, was performed to assess the safety and efficacy of autologous mesothelin‐targeted CAR T cells [32]. The results indicated that of 21 patients with MPM, none experienced on‐target, off‐tumor toxicity. Two patients had complete metabolic response, five had a partial response, and four had stable disease following treatment. While these studies highlight key improvements in CAR T treating solid tumors, more studies would be helpful in generating evidence for or against this treatment and approach. However, more rigorous evaluation is necessary to see their potential long‐term feasibility. A summary of the current trials evaluating CAR T cell targets in lung cancer is included in Table 1.

**TABLE 1:** Non‐comprehensive list of ongoing CAR T cell therapy studies in solid organ malignancies*

Cancer type	NCT number	Recruiting status	Brief title	Cell target
Brain Cancers (Glioblastoma)	NCT02208362	Ongoing Phase I	Genetically Modified T‐cells in Treating Patients with Recurrent or Refractory Malignant Glioma	IL13Ra2
	NCT03726515	Ongoing Phase I	CART‐EGFRvIII + Pembrolizumab in GBM	EGFRvIII
	NCT01454596	Completed recruiting/ phase I	CAR T Cell Receptor Immunotherapy Targeting EGFRvIII for Patients with Malignant Gliomas Expressing EGFRvIII	EGFRvIII
	NCT01109095	Completed recruiting/ phase I	CMV‐specific Cytotoxic T Lymphocytes Expressing CAR Targeting HER2 in Patients With GBM	HER2
Gastrointestinal Cancers	NCT01373047	Completed recruiting/ phase I	CEA‐Expressing Liver Metastases Safety Study of Intrahepatic Infusions of Anti‐CEA Designer T Cells	CEA
	NCT03682744	Ongoing phase I	CAR‐T Intraperitoneal Infusions for CEA‐Expressing Adenocarcinoma Peritoneal Metastases or Malignant Ascites (IPC)	CEA
	NCT01897415	Completed recruiting/ phase I	Autologous Redirected RNA Meso CAR T Cells for Pancreatic Cancer	Mesothelin
	NCT03323944	Ongoing phase I	CAR T Cell Immunotherapy for Pancreatic Cancer	Mesothelin
	NCT03159819	Ongoing phase I	Clinical Study of CAR‐CLD18 T Cells in Patients with Advanced Gastric Adenocarcinoma and Pancreatic Adenocarcinoma	Claudin 18.2
	NCT02744287	Ongoing phase I	Safety and Activity Study of PSCA‐Targeted CAR‐T Cells (BPX‐601) in Subjects with Selected Advanced Solid Tumors	PSCA
Renal Cancer	N/A	Preclinical		Carboxy‐anhydrase IX (CAIX)
	NCT03393936	Ongoing phase I	Safety and Efficacy of CCT301 CAR‐T in Adult Subjects with Recurrent or Refractory Stage IV Renal Cell Carcinoma	AXL
Prostate Cancer	NCT03089203	Ongoing phase I	CART‐PSMA‐TGFβRDN Cells for Castrate‐Resistant Prostate Cancer	PSMA
	NCT03873805	Ongoing phase I	PSCA‐CAR T Cells in Treating Patients With PSCA+ Metastatic Castration Resistant Prostate Cancer	PSCA
Ovarian Cancer	NCT03585764	Ongoing phase I	MOv19‐BBz CAR T Cells in aFR Expressing Recurrent High Grade Serous Ovarian, Fallopian Tube, or Primary Peritoneal Cancer	Folate receptor‐alpha
	NCT02498912	Ongoing phase I	Cyclophosphamide Followed by Intravenous and Intraperitoneal Infusion of Autologous T Cells Genetically Engineered to Secrete IL‐12 and to Target the MUC16ecto Antigen in Patients with Recurrent MUC16ecto+ Solid Tumors	MUC16
	NCT02792114	Ongoing phase I	T‐Cell Therapy for Advanced Breast Cancer	Mesothelin
	NCT02442297	Ongoing phase I	T Cells Expressing HER2‐specific Chimeric Antigen Receptors (CAR) for Patients with HER2‐Positive CNS Tumors	HER2
	NCT03696030	Ongoing phase I	HER2‐CAR T Cells in Treating Patients with Recurrent Brain or Leptomeningeal Metastases	HER2
	NCT04020575	Ongoing phase I	Autologous huMNC2‐CAR44 T Cells for Breast Cancer Targeting Cleaved Form of MUC1	MUC1
Thoracic Cancer	NCT02414269	Ongoing phase 1	Malignant Pleural Disease Treated with Autologous T Cells Genetically Engineered to Target the Cancer‐Cell Surface Antigen Mesothelin	Mesothelin
	NCT0305429	Ongoing phase 1	CAR T Cells in Mesothelin Expressing Cancers	Mesothelin
	NCT02706392	Ongoing phase 1	Genetically Modified T‐Cell Therapy in Treating Patients with Advanced ROR1+ Malignancies	ROR1

* Last updated on 09/29/ 2021 from clinicaltrials.gov.

### 10.1 Mesenchymal tumors

Despite a lack of specific targetable molecules, osteosarcoma is another category of solid tumors where treatment with CAR T is under investigation. Treatment has been based on widely expressed antigens across tumor types such as HER2, EGFR, and GD2 [33]. Specifically, HER2‐positive osteosarcoma cells have been identified through immunohistochemistry and flow cytometry. This discovery was followed by using HER2‐specific CAR T in xenotransplantation mice, where treatment induced tumor regression and increased survival [34].

Numerous clinical trials have since emerged to investigate CAR T cell utility for treating osteosarcoma. NCT02107963 is a completed phase I trial of a third generation anti‐GD2‐CAR used to treat GD2+ tumors in children and young adults (NCT02107963). The trial was not restricted to osteosarcomas; children with GD2+ sarcomas, neuroblastomas, and melanomas were also selected for this trial. In total, 15 patients were selected and placed in a non‐randomized manner into one of two trial arms. Both arms were treated with the same lymphodepletive regimen of cyclophosphamide 1800 mg/m^2^/d × 2 days. The first arm investigated dose‐escalation, while the second arm investigated dose expansion. Results from this trial have not been released. Six other clinical trials are currently recruiting; three are not currently recruiting. Another clinical trial, NCT01953900, is active, but no results have been published (NCT01953900).

Another phase I trial included a pediatric patient with refractory metastatic rhabdomyosarcoma that was featured in a case study discussing the child's response to multiple cycles of HER2 CAR T [35]. The patient's cancer cells had confirmed expression of HER2 on both the primary tumor and bone marrow metastasis and was enrolled in a clinical trial for patients with advanced sarcoma. A CAR T product targeting HER2 was containing a majority of CD8+ T cells. This product was first administered 4 weeks after a washout and recovery from prior chemotherapy followed by three subsequent lymphodepletions with cyclophosphamide and fludarabine prior to HER2 CAR T infusions 10 weeks apart. Following induction, morphologic and imaging studies demonstrated the absence of disease. The patient continued to receive HER2 CAR T cells without lymphodepletive therapy every 10 weeks for 6 months for disease consolidation. The child had confirmed bone marrow relapse 6 months following the last administered CAR T dose and was re‐enrolled in the trial, receiving the same lymphodepletive HER2 CAR T regimen given the maintained HER2 expression. Pembrolizumab was also started 2 weeks after the second HER2 CAR T administration and was given every 3 weeks to promote CAR T function. The patient was able to obtain a second remission that has lasted.

## 11 CHALLENGES AND OPPORTUNITIES

Contrasting with the successes of CAR T treating hematologic malignancies, the development of CAR T therapy in solid tumors has progressed at a slower pace. Challenges unique to solid tumor settings arise in the form of tumor histopathological characteristics, lack of tumor‐specific antigens, immunosuppressive tumor microenvironments (TME), and on‐target, off‐tumor toxicity that can be life‐threatening [36, 37, 38, 39].

### 11.1 Antigen specificity

The lack of the specificity of antigens to target tumor cells is a critical issue leading to on‐target, off‐tumor toxicity in solid tumors. Tumor‐associated antigens (TAA), antigens overexpressed on tumor cell surfaces, were initially thought to be an excellent target for the CAR T but utilization led to damage of normal healthy tissues throughout the body where these antigens were also present ([39],[40]). These events can be fatal. For example, one case arose when a patient treated with anti‐HER2 CAR T for metastatic colon cancer died five days later after the CAR T cells attacked healthy HER2 expressing lung epithelial cells [41]. Another case occurred when patients with neuroblastoma were given high affinity‐GD2 CAR T that attacked healthy brain tissue expressing low levels of GD2, causing fatal encephalitis [41]. Both cases highlight the detriment of on‐target off‐tumor toxicity while also demonstrating the need for better targeting antigens.

Another differing factor between hematologic and solid malignancies is the homogeneity of antigens presented. Hematologic malignant cells tend to express homogeneous TAA, but solid tumors display antigen heterogeneity between tumor types and the primary versus metastatic stages of individual tumors ([40],[42]). This means that one group of tumor cells expressing the antigen used for targeting would be destroyed, but other groups of tumor cells lacking the same antigen would escape and continue proliferating.

### 11.2 Tumor microenvironment

Vital to CAR T cells eliminating solid tumors is the proper trafficking of CAR T cells to the surface of the cancer so that they may bind to the target protein, but the TME impedes this transit. Solid tumors produce chemokines like CXCL1, CXCL12, and CXCL5 within the TME, preventing T cells from reaching the tumor cells. An example specific to CXCL12 revolved around a study in pancreatic cancer. CXCL12 is produced by carcinoma‐associated fibroblasts (CAF) expressing fibroblast activation protein (FAP) [43]. Tumor cells have high concentrations of CXCL12 suspected to be due to the overexpression of high mobility group box 1 (HMGB1), an overexpressed protein by metabolically stressed cancer cells that captures CXCL12. T‐cells were less abundant in areas where FAP+ cells were present, with further investigation demonstrating an association with CXCL12. Other chemokines like CXCL5 secreted by solid tumors recruited CXCR2‐expressing myeloid‐derived suppressor cells (MDSCs) to the tumor microenvironment that secrete cytokines and enzymes suppressing local T cells activation and viability [44].

Other factors within the microenvironment including excessive blood vessels, fibroblasts, and myeloid cells producing extracellular matrix, also serve to impede CAR T trafficking ([38],[40],[42]). Other immune repressor cells are recruited to the TME such as regulatory T cells (Treg) and tumor‐associated macrophages that act similarly to MDSCs by preventing cytotoxic cells from killing tumor cells. Immunosuppressive cytokines including TGF‐β and PD‐L1, a checkpoint inhibitor protein, are overproduced downregulating CD8 T cell action and promote Treg maturation ([38],[40]).

### 11.3 Future directions

Each of these hurdles present in solid tumors contributes to the limited success of CAR T against solid tumors. Still, attempts are being made to overcome some of these obstacles, mainly through the design of CAR T agents. One strategy being investigated to overcome the issue of on‐target, off‐tumor toxicity from TAAs is the engineering of CARs targeting glycopeptide epitopes from mutations creating glycosylation present on tumor cells. However, this area of interest still requires extensive testing to ensure damage does not occur in healthy tissues [37]. Another idea is to use a strategy called Boolean AND‐gate logic where multiple receptors are engineered on the T cells so that activation requires specific combinations of signals that will not be present beyond the TME [45].

In a similar vein to Boolean AND‐Gate logic, AND‐NOT logic is another strategy where CAR specificity could be increased by triggering T cell activation only in the presence of a TAA and not in the presence of a second antigen expressed on healthy cells. This can be achieved through engineering CARs to express a zipCAR, a universal receptor, consisting of a leucine zipper ectodomain fused to the transmembrane and intracellular signaling domains. Split, universal, programmable CAR T products (SUPRA CARS) utilize zipCARs [45]. ZipCARs lack ligand‐binding domains and must be reconstituted with exogenous zipFv proteins, single‐chain variable fragment adaptors, to activate T cells and bind with TAAs. It is thought that the second class of zipFv molecules could be administered to a patient to compete against zipCAR for binding, effectively preventing CAR‐T activation in normal tissues.

These methods are not without drawbacks. There is the possibility that solid tumor cells may escape detection due to the various stages of mutation a tumor may be in preventing recognition by CAR‐T products requiring combinations of antigens for activation. Mutations for glycosylation may also be absent eliminating one method of differentiation between normal and solid tumor cells [37]. To avoid convoluted engineering practices, the simplest way to overcome a poor antigen choice is to select a better antigen specific to the target, but this too poses its problems. An example is EGFRvIII found in glioblastomas. Studies initially showed that it might serve as a good target, but heterogeneity in expression and tumor response to downregulate EGFRvIII leads to marginal growth effects on the tumor [37].

## 12 CONCLUSION

While the investigation of CAR T cell therapy in solid tumors is relatively new, an extensive amount of ongoing research will be necessary to assess the safety and feasibility of their place in treatment in solid tumors. Most of the current clinical studies discussed in this article are phase 1 trials that mainly investigate CAR T products' safety and efficacy in solid tumors with several in the pipeline. Indeed, the need to better understand these therapies will continue to exist and develop over time. CAR T therapy has great potential to impact the entire landscape of solid tumor malignancies just as it has for hematological cancers. Perhaps, the success of these current trials will lead to the progress of future phases 2 and 3 trials. In addition, the new designs for CAR T products emerging may serve to circumvent challenges posited by the solid tumor microenvironment. As research expands in CAR T therapies, further challenges and opportunities will continue to emerge. Thus, scientific research and development will continue to grow, guide, and impact its potential in a positive direction.
